# Childhood vaccine refusal in Türkiye: a dual perspective qualitative study of parents and healthcare workers

**DOI:** 10.3389/fpubh.2026.1896557

**Published:** 2026-09-07

**Authors:** Esra Sezer, Yesim Isil Ulman

**Affiliations:** 1 Department of Nursing, Faculty of Health Sciences, Acibadem Mehmet Ali Aydinlar University, Istanbul, Türkiye; 2 Bioethics MSc Programme, Institute of Health Sciences, Acibadem Mehmet Ali Aydinlar University, Istanbul, Türkiye; 3 Department of History of Medicine and Ethics, School of Medicine, Acibadem Mehmet Ali Aydinlar University, Istanbul, Türkiye

**Keywords:** childhood immunization, parental autonomy, public health ethics, qualitative research, vaccination hesitancy, vaccination refusal

## Abstract

**Background:**

Childhood vaccine refusal is a growing public health challenge worldwide. In Türkiye, formal refusal, defined as a parental decision to decline one or more scheduled vaccines that is documented in primary care immunization records, rose from 183 families in 2011 to more than 12,000 in 2016, yet the structural, cultural, and ethical dimensions of parental decision-making remain poorly understood. This study explored these tensions from the perspectives of both vaccine-refusing parents and vaccine-administering healthcare workers.

**Methods:**

Semi-structured interviews were conducted between February and August 2017, before the COVID-19 pandemic, with 19 vaccine-refusing parents and 21 healthcare workers (physicians, nurses, and midwives) in Türkiye. Participants were recruited through criterion-based purposive sampling combined with snowball sampling, an approach appropriate for a hard-to-reach group. Data were analyzed using hybrid inductive–deductive thematic analysis, combining a deductive framework derived from the interview guides with inductively generated codes. Themes were then interpreted through principlism, relational autonomy, and the best interest of the child standard.

**Results:**

Four interconnected ethical tensions were identified: (1) the gap between informed refusal and uninformed choice, shaped by information asymmetry and structural constraints within the performance-based healthcare system; (2) a crisis of trust in healthcare institutions, which participants associated with financial incentives in the payment model and with distrust of the pharmaceutical industry; (3) conflict between religious and medical authority over vaccine permissibility; and (4) contested parental autonomy within patriarchal family structures where mothers’ voices were frequently marginalized.

**Conclusion:**

Vaccine refusal in Türkiye is embedded in structural, cultural, and relational conditions that information provision alone cannot address. Participants in both groups described the performance-based payment model, which ties part of physicians’ pay to preventive service targets, as favouring documentation over dialogue. Effective strategies may therefore require reform of this model, religious literacy training, gender-sensitive clinical approaches, and collaboration with trusted community leaders. These pre-COVID-19 findings provide a baseline for understanding the persistent roots of vaccine hesitancy.

## Introduction

Childhood vaccination is widely recognized as one of the most significant public health achievements of the past century; modelling of five decades of the Expanded Programme on Immunization attributes at least 154 million averted deaths to vaccination since 1974 (1). Declines in vaccination coverage, however, have led to the resurgence of vaccine-preventable diseases in multiple countries, with measles outbreaks serving as a stark reminder that population-level immunity cannot be sustained when a critical minority of parents refuse vaccination for their children (2). Yet despite the overwhelming scientific consensus on vaccine safety and efficacy, the refusal of childhood vaccination by parents has emerged as a growing global challenge (3, 4). In 2019, the World Health Organization identified vaccine hesitancy as one of the top 10 threats to global health (5); the term is defined by the SAGE Working Group as delay in acceptance or refusal of vaccines despite the availability of vaccination services (6). This tension between the demonstrated benefits of vaccination and the persistent refusal by a minority of parents raises fundamental questions that extend well beyond epidemiology into the domain of bioethics: Where does parental autonomy end and the obligation to protect the common good begin? Whose interests should prevail when the health of a child, who cannot consent for themselves, is at stake?

These questions are far from abstract. The ethical dimensions of vaccine refusal involve a constellation of competing principles: respect for parental autonomy, the best interest of the child, the duty of beneficence owed by healthcare professionals, and the collective responsibility to maintain herd immunity (7, 8). The principlist framework, while foundational, does not resolve these tensions; it merely maps them (7). To understand how these ethical tensions manifest in lived experience, empirical inquiry is essential. As Borry et al. (9) argued in their account of the “empirical turn” in bioethics, normative analysis gains depth and credibility when grounded in the perspectives of those who navigate ethical dilemmas in practice.

Türkiye provides a particularly instructive context for examining the ethics of childhood vaccine refusal. Although vaccination is not legally mandatory in Türkiye, the national Expanded Program on Immunization covers 13 childhood diseases and is delivered primarily through the family physician system (10). Within this system, a performance-based payment model ties part of physicians’ remuneration to the attainment of preventive service targets, including vaccination coverage rates; family physicians who do not meet these targets face deductions from their contracted payment and must document an explanation for every unmet preventive service, including each missed vaccine dose (11, 12). This arrangement creates a structural incentive that shapes the clinical encounter in ways that have ethical implications of their own. Despite historically high immunization rates, Türkiye has experienced a marked rise in vaccine refusal in recent years. The number of families formally refusing childhood vaccination increased from 183 in 2011 to 980 in 2013, reaching 5,400 in 2015 and exceeding 12,000 by 2016 (13, 14). This escalation coincided with the proliferation of anti-vaccine content on social media platforms and the influence of alternative health practitioners and religious figures who discouraged vaccination (13, 14). It also followed a landmark ruling of the Constitutional Court of the Republic of Türkiye in November 2015, which held that administering vaccines to a child over parental objection lacked an adequate legal basis and therefore violated the constitutional guarantee of physical integrity (15). The decision was reported in the national press and on social media as a victory for refusing parents, and subsequent analyses have identified it as a turning point in the trajectory of refusal in Türkiye (13, 16).

The existing literature on vaccine refusal in Türkiye is predominantly quantitative. Cross-sectional studies have measured the prevalence of parental vaccine hesitancy, identified sociodemographic predictors, and assessed the impact of the COVID-19 pandemic on vaccination attitudes (17–20). However, quantitative approaches capture the extent of the phenomenon without illuminating the moral reasoning, experiential logic, and relational dynamics that underpin parental decisions. Qualitative studies that explore these dimensions remain scarce in the Turkish context, though such approaches have yielded important insights in other settings (21). A notable exception is the work of Yalçin et al. (22), which examined healthcare professionals’ observations regarding parental vaccine refusal through qualitative methods, but did not include the voices of vaccine-refusing parents themselves. To our knowledge, no published study from Türkiye has employed a dual-perspective qualitative design to explore the ethical dimensions of vaccine refusal from the standpoints of both vaccine-refusing parents and vaccine-administering healthcare workers simultaneously.

This gap is significant because the ethical tensions surrounding vaccine refusal are not experienced by parents alone. Healthcare workers who administer vaccines occupy a morally complex position that gives their accounts distinctive evidentiary value for an ethical analysis. First, they are the only participants positioned to describe the institutional constraints (consultation time, documentation requirements, and performance targets) that shape whether an informed discussion is possible at all; parents can report that counselling was inadequate, but not why. Second, because they encounter many refusing families rather than one, they can identify recurrent patterns, such as the intergenerational and gendered distribution of decision-making authority, that individual parents experience as simply the way their own household works. Third, they are themselves subject to competing obligations, namely professional duties of beneficence and non-maleficence on one hand and respect for parental authority on the other, as well as the duty to prevent harm to third parties when refusal threatens herd immunity (8), so their accounts expose the ethical tension from the side of the clinician rather than the family. They serve as what Giddens (23) termed “access points” to abstract expert systems, the healthcare system, the scientific establishment, and their interactions with parents can either build or erode the trust upon which vaccination programs depend (24, 25). Understanding vaccine refusal as an interactional phenomenon, shaped by the encounter between parents and healthcare professionals within specific structural and cultural contexts, therefore requires hearing both voices.

The data presented in this article were collected through qualitative interviews conducted in Türkiye in 2017, before the COVID-19 pandemic fundamentally altered public discourse on vaccination worldwide. While this temporal distance might appear to be a limitation, we argue that it constitutes a distinctive contribution. The pre-pandemic data capture the landscape of childhood vaccine refusal in Türkiye at a critical inflection point, when refusal rates were rising sharply and before the pandemic-era politicization of vaccines and the volume of COVID-19 misinformation that subsequently reshaped public attitudes (26, 27). They therefore document an earlier configuration of the phenomenon against which pandemic-era accounts can be compared, rather than a purer or more valid one. Recent post-pandemic studies from Türkiye indicate that the core themes identified in the pre-pandemic period, distrust in healthcare institutions, susceptibility to misinformation, religious motivations, and family power dynamics, have not only persisted but intensified (17, 19). This continuity suggests that the roots of vaccine refusal are structural and cultural rather than merely reactive, and that pre-pandemic empirical data offer a valuable baseline against which pandemic-era transformations can be understood.

This study aims to explore the ethical dimensions of childhood vaccine refusal in Türkiye through an empirical bioethics approach. By examining the perspectives of both vaccine-refusing parents and vaccine-administering healthcare workers, it seeks to illuminate the moral reasoning, relational dynamics, and structural conditions that shape vaccine refusal in a specific cultural context. In doing so, it contributes to the growing body of scholarship that brings ethical analysis into dialogue with the lived experiences of those who navigate complex health decisions, offering evidence-based insights for the design of more effective and equitable vaccination programs (28).

## Materials and methods

### Study design and approach

This qualitative study employed semi-structured interviews within an empirical bioethics framework (9, 28). Empirical bioethics brings together empirical social research and normative ethical analysis, using qualitative data not merely to describe but to inform and refine ethical inquiry. In this study, thematic analysis of interview data generated descriptive findings, which were subsequently interpreted through the lens of bioethical principles, specifically, autonomy, beneficence, non-maleficence, justice (7), relational autonomy (29, 30), and the best interest of the child standard. These three frameworks were selected because each addresses a limitation of the others in this particular context: principlism identifies the competing obligations in play but treats the deciding agent as given; relational autonomy interrogates that assumption by asking how the capacity to decide is enabled or constrained by social position; and the best interest of the child standard expresses the beneficence owed to the child, who is affected by the decision but takes no part in making it. This two-stage analytical process, moving from empirical description to normative interpretation, reflects what Davies et al. (31) describe as an integrated empirical-normative approach. Data were collected in Türkiye between February and August 2017.

### Participants and recruitment

Two participant groups were recruited using criterion-based purposive sampling supplemented by snowball sampling (32):

Vaccine-refusing parents (*n* = 19): The single eligibility criterion was the phenomenon of interest itself: being the parent of a child aged zero to 6 years for whom some or all routine childhood vaccinations had been declined. Refusal status was established by participant self-report during eligibility screening and confirmed in the opening minutes of each interview. Some participants had signed a formal refusal form in primary care; others had declined vaccines without a documented refusal being recorded. Having signed a formal refusal form was deliberately not adopted as an inclusion criterion, since restricting the sample to formally documented refusers would have excluded parents who avoided the clinical encounter altogether, a group whose disengagement from primary care is itself of ethical interest. Whether refusal was partial (some vaccines declined) or complete emerged during the interviews rather than at recruitment and, given the sample size, was not used as a stratifying variable; the analysis therefore treats refusal as a single category. Semi-structured interviews were conducted face-to-face at locations chosen by the participants, including their workplaces and quiet public spaces. Three participants were fathers; in each of these cases, the mothers had declined to participate. Among the remaining 16 female participants, ages ranged from 33 to 41 years, with varying levels of education. Data collection continued until successive interviews ceased to generate new codes relevant to the study aims.

No further *a priori* selection criteria were imposed, and maximum variation was not sought as a formal sampling strategy. This reflects the exploratory aim of the study and the difficulty of reaching a socially stigmatized population. Parents who refuse vaccination often expect criticism from clinicians and from their own social circles, and are therefore reluctant to identify themselves to researchers. Snowball sampling was adopted for this reason, since referral by a trusted peer was in practice the only reliable route of access, and recruitment was additionally extended through social media groups in which vaccine-refusing parents were active. The diversity ultimately present in the sample, in education, age, and stated grounds for refusal, is therefore best understood as an emergent property of the recruitment chains rather than as a designed feature. For the same reason, we have avoided the language of data saturation in reporting the point at which data collection ceased for either participant group: saturation implies that the range of the phenomenon has been exhausted, a claim that sits uneasily with a sample not constructed for maximum variation, and its applicability to thematic analysis has in any case been questioned (33). We therefore report the pragmatic criterion actually applied: interviewing continued until successive interviews no longer generated codes relevant to the study aims.

Healthcare workers (*n* = 21): Eligibility required current, direct responsibility for administering childhood vaccines. Within this criterion, recruitment deliberately spanned the three professional groups involved in vaccine delivery in Turkish primary care, that is, physicians (family physicians and paediatricians), nurses, and midwives, because these roles differ in the nature and duration of their contact with families and were therefore expected to yield different vantage points on the refusal encounter. This was the one respect in which sampling was purposively structured in advance. Participants were recruited from primary care settings in Istanbul (*n* = 17) and Bursa (*n* = 3), with one participant from a hospital setting. Snowball sampling was used, with initial participants recommending colleagues; this was appropriate given that access to clinicians during working hours depended in practice on collegial introduction. Istanbul was selected as the principal recruitment site because it is where the first author was based and because its size and internal heterogeneity encompass a wide range of primary care settings; the Bursa participants were reached through snowball sampling, as referral chains extended beyond Istanbul. Given the small number of participants outside Istanbul, no comparison between the two provinces was undertaken, and the accounts were analyzed as a single corpus; this constraint is revisited in the limitations. The group comprised 14 women and seven men, aged 25 to 54 years, with professional experience ranging from two to 28 years. All healthcare worker interviews were conducted face-to-face in participants’ workplaces, typically during lunch breaks to minimize disruption. One interview was excluded because the participant did not consent to audio recording. In line with qualitative research practice, data collection ceased when further interviews no longer yielded new codes.

Participant age, sex, educational level, and professional role are reported throughout the findings because they situate each account within the social relations the analysis is concerned with, most directly for the fourth theme, in which generational position and gender are constitutive of decision-making authority rather than incidental to it. These attributes were not used to stratify the sample, to structure the coding frame, or to compare subgroups, and no claim is made that any theme is characteristic of a demographic category. They function as contextual information supporting the reader’s assessment of transferability.

### Data collection

Two separate semi-structured interview guides were developed following a review of the literature on vaccine refusal: one for parents (eight thematic areas including reasons for refusal, vaccine-specific choices, information sources, decision-making dynamics, and disease perceptions) and one for healthcare workers (four thematic areas including observations on parental refusal, communication challenges, systemic factors, and policy perspectives). Questions were designed to be open-ended and non-directive, allowing participants to express their views in their own terms (34). Emergent topics raised by participants were incorporated into subsequent interviews.

All interviews were audio-recorded using a digital voice recorder (Sony ICD-PX470), with a telephone backup. Interview duration ranged from 33 to 72 min (mean: 43 minutes) for healthcare workers and from 31 to 68 min (mean: 45 minutes) for parents. All interviews were conducted in Turkish by the first author.

### Data analysis

Interviews were transcribed verbatim by the first author. Data analysis was conducted following the completion of data collection, using thematic analysis as described by Braun and Clarke (35), operationalized as a hybrid inductive–deductive process of the kind set out by Fereday and Muir-Cochrane (36).

The deductive component consisted of an *a priori* template of codes derived from the structure of the interview guides, in which main interview questions served as provisional themes and follow-up questions as provisional sub-themes. This template provided an initial organizing structure for the first pass through the transcripts. The inductive component operated from the first transcript onward: segments of data that did not correspond to any template code were coded openly and in participants’ own terms, and these data-driven codes were added to the working code list as they arose. Several of the study’s central findings originated inductively rather than from the template, most notably the role of mothers-in-law and other extended-family figures in vaccine decisions, and healthcare workers’ accounts of the performance system as a constraint on counselling, neither of which had been anticipated in the interview guides.

The two streams were integrated at the stage of theme development rather than kept separate. Once coding was complete, the full code list for each participant group was reviewed as a whole, without regard to whether a given code had originated deductively or inductively, and codes were clustered into candidate themes on the basis of shared meaning. Where an inductive code proved more analytically productive than the template code under which it had initially been filed, the template category was subordinated to it or dissolved. Separate code tables were maintained for parents and healthcare workers to preserve the distinctive perspective of each group while enabling cross-group comparison at the theme level; the four themes reported here are those for which both groups contributed substantive accounts.

For the purposes of this article, the descriptive themes were subsequently re-examined through a bioethical lens. The analytical focus shifted from “what do participants report?” to “what ethical tensions are embedded in these reports?” This interpretive step, moving from empirical findings to normative analysis, represents the distinctive contribution of the empirical bioethics approach adopted in this study. Four interconnected ethical tensions were identified through this process: information asymmetry and the question of informed refusal, the crisis of trust in the clinical relationship, the conflict between religious and medical authority, and contested autonomy within family power dynamics. Interviews were analyzed in the original language (Turkish); selected quotations presented in the findings were translated into English by the first author and verified by a bilingual colleague.

### Ethical considerations

Ethical approval was obtained from the Acıbadem University and Acıbadem Healthcare Institutions Medical Research Ethics Committee (ATADEK; approval date: 16 February 2017, decision number: 2017-3/35). Written informed consent was obtained from all participants prior to interviews, in accordance with the Declaration of Helsinki. Participants were informed of their right to withdraw at any time and to request that audio recording be paused. Anonymity was ensured through the use of participant codes (e.g., P1 for parents, HW1 for healthcare workers) in all transcripts and publications. No participant requested withdrawal during or after the interview process.

### Researcher positionality and reflexivity

The first author, who conducted all interviews, holds a PhD in Nursing Education and a Master’s degree in Bioethics. At the time of data collection, she was a registered nurse working in a neonatal intensive care unit. This dual background, combining clinical nursing practice with formal training in bioethics, informed both the design of the study and the analytical lens applied to the data. Her clinical role provided familiarity with vaccination practices and the healthcare system, while her bioethics training sensitized her to the ethical dimensions of participants’ accounts. However, this positioning also meant that she was perceived by some parent participants as a representative of the healthcare system from which they had disengaged. This dual identity was disclosed to all parent participants prior to interviews. The researcher maintained a reflexive stance throughout data collection and analysis, remaining attentive to the ways in which her professional identity might shape the interview dynamic and the interpretation of data. Notably, parent participants did not appear to be inhibited by this disclosure; on the contrary, several expressed appreciation for the opportunity to share their perspectives with a healthcare professional willing to listen without judgment. Her professional background also facilitated access to and rapport with healthcare worker participants.

The second author is a professor of bioethics and medical history with extensive expertise in the ethical dimensions of healthcare practice. She has been serving as a member of the Bioethics Monitoring Committee of the UNESCO National Commission since 2022. Her role in this study involved oversight of the research design, critical review of ethical interpretations, and ensuring the analytical rigour of the normative dimensions of the findings.

### Trustworthiness

The trustworthiness of the study was addressed through several strategies aligned with established criteria for qualitative rigour (37). Credibility was enhanced through prolonged engagement with participants, triangulation of perspectives across two stakeholder groups, and the use of verbatim transcription. Transferability was supported through thick description of the research context, participant characteristics, and the Turkish healthcare system. Dependability was maintained through a transparent audit trail documenting the coding process and analytical decisions, including the provenance of each code as template-derived or data-derived. Confirmability was strengthened through reflexive practice and ongoing critical dialogue between the authors regarding emerging interpretations. The reporting of this study follows the Consolidated Criteria for Reporting Qualitative Research (COREQ) checklist (38).

## Results

Four interconnected ethical tensions emerged from the analysis of interviews with vaccine-refusing parents and vaccine-administering healthcare workers. Table 1 presents the thematic structure with representative quotations from both participant groups. Each theme is presented below through the accounts of both groups; their interpretation in the light of bioethical theory and the wider literature is developed in the Discussion.

**Table 1:** Themes, sub-themes, and supporting quotations.

Theme/sub-theme	Representative quotations
Informed refusal or uninformed choice?	
1a. Perceived inadequacy of information from healthcare providers	“Nobody in a position of authority has been able to answer questions such as: which vaccines are truly necessary, what happens if they are not administered, and what are their ingredients? Frankly, I believe there is no expert on this subject in Türkiye.” (P3, male, 30, postgraduate)
	“The Community Health Center kept calling and sending people to our door, but nobody gave any concrete information. The paediatrician offered no real knowledge, just accusations.” (P2, female, 29, university)
	“The performance system exists. Vaccinations are deducted from it. When a family refuses, we have to report it. But I cannot say that every colleague makes an equal effort to inform them.” (HW10, female, 25, physician)
	“Nobody truly takes ownership of it. There is no sense of taking responsibility for patients. I think people withdraw because they cannot do what they want to do.” (HW21, female, 27, physician)
1b. Social media as primary information source	“During my pregnancy, while researching vaccine ingredients online, I came across comments from parents who had observed side effects. When I told my husband, we jointly decided not to vaccinate our child.” (P5, female, 30, secondary school)
	“There is a social media group for unvaccinated families. Some have experienced harm; others simply chose not to vaccinate. They select members carefully because a previous group was shut down after complaints.” (P8, female, 28, university)
	“Especially on internet forums, they say ‘I read this.’ They do not read what they should, they read nonsense written by random people. There are very large online groups.” (HW3, female, 46, physician)
	“Unfortunately, I attribute much of it to technology, in this system where we can easily access information but struggle to distinguish right from wrong, misinformation has multiplied.” (HW6, female, 29, nurse)
The crisis of trust in the clinical relationship	
2a. Distrust of pharmaceutical industry and health system	“Vaccines are a huge source of revenue. The health sector treats patients as customers. They damage our health with drugs and vaccines and then promise to fix it with more drugs, a vicious cycle.” (P8, female, 28, university)
	“If objective medical circles reach a consensus, and if the conditions of production and access change, then I would reconsider my decision. The swine flu episode shattered our trust.” (P2, female, 29, university)
	“People’s trust has been shaken for several reasons. When families refuse, there are many factors, social media, the things they read, distrust of the pharmaceutical industry.” (HW5, male, 42, physician)
2b. Anti-vaccine physicians reinforcing parental distrust	“She was a very organic-minded doctor. She told me: ‘Go online and read about the ingredients, read both sides, then decide for yourself. But if it were me, I would not vaccinate my child.’” (P3, female, 50, doctoral degree)
	“Some paediatricians oppose vaccination. Because they are doctors, families trust them more when they warn about neurological damage.” (HW12, female, 34, nurse)
	“Such individuals’ professional credentials should be questioned. I cannot imagine a paediatrician being against vaccination.” (HW4, male, 48, physician)
Religious authority versus medical authority	
3a. Vaccine ingredients perceived as religiously impermissible	“We believe that vaccines contain substances that are not halal, such as pig and monkey DNA. We are not vaccinating for this reason. However, if the state produces vaccines that respect halal requirements and convinces us of their necessity, we could reconsider.” (P3, male, 30, postgraduate)
	“I did not allow her to eat or drink any packaged product without a halal certificate. I was careful even during pregnancy. I first learned that vaccines are not halal from certain faith-based online platforms.” (P8, female, 25, secondary school)
3b. Fatalism and divine will	“If God has written that it will happen, no matter what I do, the disease will come. If He has not written it, it will not come.” (P4, female, 36, university)
	“When it suits us, we say ‘God will protect’ but then we rush to get vaccinated before the disease arrives, trusting the vaccine’s protection instead.” (P8, female, 28, university)
3c. Healthcare workers’ challenges with religiously motivated refusal	“Those who refuse for religious reasons, we cannot change them. We can sometimes persuade the others, but not those who refuse on religious grounds.” (HW14, male, 26, physician)
	“There is a religious dimension to vaccine refusal, especially families who believe the content is pork-derived and see it as religiously forbidden.” (HW10, female, 25, physician)
	“They say their religious leader has told them it is not permitted. They say their community does not allow it, and they are not open to further discussion.” (HW15, male, 30, physician)
Whose autonomy? Family power dynamics	
4a. Mothers silenced by patriarchal family authority	“Even during childbirth, the mother-in-law always outweighed the husband. She had more say, intervened in everything. The same system continues when it comes to vaccination.” (HW8, female, 26, midwife)
	“A grandmother had heard somewhere that vaccines harm children. Because it was a male grandchild, she told her daughter-in-law not to vaccinate, and the daughter-in-law complied.” (HW1, female, 31, nurse)
	“In the east, it is more the father. In the city it seems more equal. But in rural areas and certain neighborhoods, it is the father.” (HW21, female, 27, physician)
4b. Mothers as autonomous decision-makers	“I got a lot of backlash, people looked at me as if I were an alien. My mother spoke to my husband, trying to get him to convince me. I told him: ‘Whether the child gets sick or not, I am the one who will deal with it. Do not interfere.’ He calls me ‘Hippocrates.’” (P5, female, 31, university)
	“My husband is pro-vaccine, pro-doctor. The hardest part was convincing him. To be honest, he was not convinced, he just had to accept it.” (P10, female, 32, secondary school)
	“I read and believed I was making the right decision. My husband and I decided together. The moral dimension was very difficult, but the more information I gathered, the more at ease I felt.” (P2, female, 29, university)

Quotations were originally collected in Turkish and translated into English by the first author. Participant codes indicate group (P, parent, HW, healthcare worker), sex, age at interview, and highest level of education. All identifying information has been removed to ensure participant anonymity.

### Informed refusal or uninformed choice? The problem of information asymmetry

A central question raised by the data concerns whether parental vaccine refusal was described by participants as an informed decision or as a choice made under conditions of restricted information. Parents consistently reported that healthcare providers failed to offer adequate information about vaccine content, risks, and benefits. One mother described her experience:

I was constantly called from the Community Health Center, they sent someone to our door and had official reports drawn up but nobody gave me any concrete information. … The paediatrician said things like “you have brought trouble on yourself,” with no substance to them. (P2, female, 29, university).

Another parent explained that the information vacuum drove her to seek knowledge elsewhere, ultimately leading her to anti-vaccine social media communities:

I came across a group on social media and what I read made sense to me. They shared enough articles, cases of affected children, and current news. Statistics on allergies, autism, and immune system disorders that emerged after vaccination convinced me. … We are not adequately informed by healthcare workers about the contents of vaccines. (P7, female, 26, university).

Healthcare workers, for their part, acknowledged this information gap but attributed it to structural constraints within the healthcare system. The performance-based payment model, which ties part of physicians’ remuneration to preventive service targets including vaccination coverage, was identified as a key factor. Rather than enabling extended dialogue with hesitant parents, participants described a system that rewarded rapid encounters focused on achieving numerical targets:

I think there is less control than there should be; I do not think doctors try hard enough. This vaccine tracking system has been in place for seven years, but as I said, not everyone gives sufficient care. It might not be 100% coverage, but the fact that it always appears as 100% is suspicious… it needs better oversight. (HW17, male, 35, physician).

A doctor could show an unvaccinated person as vaccinated if they do not want the hassle. When we look at vaccination rates at family physician meetings, some report very high rates every year…I do not think that’s accurate. In all these years, has there really never been a single family who refused? It does not seem right. So the system has this vulnerability. (HW16, male, 54, physician).

The two groups thus converged on the same feature of the encounter from opposite sides: parents described receiving assertion rather than explanation, and healthcare workers described a set of institutional conditions under which explanation was difficult to deliver and easy to leave undocumented.

### The crisis of trust in the clinical relationship

Trust emerged as a central axis around which vaccine refusal revolved. Parents expressed distrust not only of individual healthcare providers but of the broader institutional architecture of vaccination, including pharmaceutical companies, governmental health agencies, and the incentive structures that link them.

Childhood vaccines are a system created by pharmaceutical companies to make money, that’s my view. … The issue is not protecting children; it is about self-interest and profit, pharmaceutical companies, the performance bonuses of doctors and midwives who administer the vaccines. The more vaccines, the more money. (P7, male, 35, secondary school).

Vaccines are a huge source of revenue. The reason they continue is capitalism…there are higher powers at play. The health sector treats patients as customers. They ruin our health with drugs and vaccines and then promise to fix it with more drugs. (P8, female, 28, university).

For some parents, this distrust was compounded by hostile and demeaning encounters with healthcare providers, which eroded whatever residual trust might have supported dialogue:

The doctor called the next day and was aggressive towards me. He used derogatory labels and made insulting assumptions about my beliefs. … There were threats like “we’ll take your child away.” … I do not trust any of them, the health center doctor, the nurse, the officials from the Public Health Center. (P8, female, 25, secondary school).

Healthcare workers recognized the trust deficit and expressed frustration at the systemic conditions that contributed to it. Several identified anti-vaccine physicians as a particularly damaging source of misinformation that compounded parental distrust:

Some paediatricians oppose vaccination. Because they are doctors, families trust them more when they warn about neurological damage from vaccines. (HW12, female, 34, nurse).

For example, Dr. Wakefield became very famous for his claims about vaccines and autism, but he was later stripped of his license and his claims were disproved by numerous studies. … I cannot imagine a physician, especially a paediatrician — being against vaccination. The professional credentials of such individuals should be questioned. (HW4, male, 48, physician).

Across both groups, accounts of distrust were directed at two distinct objects: the institutions and industries behind vaccination, and the individual clinician encountered in the consulting room. Parents who described the second frequently also described the first, and the performance system appeared in both registers, invoked by parents as evidence of financial motive and by healthcare workers as a constraint on the time available to counter it.

### Religious authority versus medical authority

For a subset of parents, vaccine refusal was grounded not in distrust of science per se but in religious conviction, specifically, the belief that vaccine ingredients are incompatible with the dietary and purity requirements of their faith.

Their contents are full of substances deemed impure by Islam. I believe it is wrong to inject things like monkey kidney cells, mouse cells, and pig derivatives into the body of a three-month-old or one-year-old baby. (P6, male, 25, university student).

I have not allowed her to eat or drink any packaged product without a halal certificate. I was careful even during pregnancy. … I first learned that vaccines are not halal from certain faith-based online platforms. (P8, female, 25, secondary school).

Others expressed a fatalistic religious worldview in which illness was understood as divinely ordained and vaccination as an unnecessary, even presumptuous, intervention:

If God has written that it will happen, no matter what I do, the disease will come. If He has not written it, it will not come. (P4, female, 36, university).

Healthcare workers identified religiously motivated refusal as the most intractable form of resistance they encountered:

Those who refuse for religious reasons, we cannot change them; we can persuade the others, but not them. The last patient we had was part of a religious community; vaccination was apparently not practiced in their community. I do not know the details. (HW14, male, 26, physician).

When I asked where she had learned these things, she let it slip during our conversation. She also had very strong religious rhetoric, she told me straight out that vaccines are haram. … As for the mother, I do not even bother speaking to her; her word carries no weight. The woman could not even express an opinion. (HW2, female, 27, nurse).

Parents in this group consistently located the source of their information outside both the healthcare system and any official religious institution, citing faith-based online platforms and local informal religious figures; none referred to a formal ruling from a recognized religious authority. The final quotation is also notable for the way religious grounds and gendered patriarchal authority appear together in a single account, a pattern taken up in the following section.

### Whose Autonomy? Family Power Dynamics and the Best Interest of the Child

The data complicate the assumption that vaccine decisions are made by a single deliberating parent. In numerous accounts, the decision was shaped, and sometimes determined, by other members of the household, with mothers’ preferences frequently subordinated.

Healthcare workers provided vivid accounts of these dynamics:

The mother is the person with the least say. We have patients where the mother comes secretly to have the vaccine administered. In some cases, the family does not want the vaccine but the mother genuinely does, she comes and says, “my father-in-law said not to, but please don’t tell them.” (HW15, male, 30, physician).

The mothers-in-law are the dominant ones in the families. They are the decision-makers, if she says vaccinate, they vaccinate; if she says do not, they do not. (HW12, female, 34, nurse).

Something like this happened, when the father was not at home, some mothers could not bring themselves to have the vaccine administered. We would wait to catch the father at home; those we could not reach, we had to get signed refusal forms from. (HW21, female, 27, physician).

The gendered nature of these dynamics was particularly evident in cases where religious authority and patriarchal family structures intersected. As noted in the preceding section, one nurse described a father whose strong religious convictions drove the refusal decision while the mother was entirely excluded from the conversation, unable even to express an opinion (HW2).

Among the parent participants, the picture was more varied. Several mothers described themselves as the primary decision-makers, having researched the topic and then persuaded, or overridden, their partners:

My husband is pro-vaccine, pro-doctor. The hardest part was convincing him. To be honest, he wasn’t convinced, he just had to accept it. (P10, female, 32, secondary school).

I got a lot of backlash. People looked at me as if I were an alien or had changed my religion. My mother was the first, she spoke to my husband, trying to get him to convince me. … I told him, “Whether the child gets sick or not, I’m the one who will deal with it. Do not interfere.” He said fine. He calls me “Hippocrates.” (P5, female, 31, university).

The two groups therefore described contrasting configurations of household authority: healthcare workers reported repeated encounters with families in which mothers were excluded from a decision they would then have to carry out, while several parent participants described having made and defended the decision themselves against family opposition. What the accounts share is that in neither configuration was the decision the product of an individual acting alone.

## Discussion

The four themes identified in this study, information asymmetry, the crisis of trust, the conflict between religious and medical authority, and contested autonomy within family power dynamics, are not isolated phenomena. They form an interconnected system in which each tension reinforces the others. Information deficits feed distrust; distrust drives parents towards alternative sources of authority, including informal religious leaders and social media communities; and patriarchal family structures further constrain the conditions under which autonomous decision-making might occur. Taken together, these findings suggest that childhood vaccine refusal in Türkiye cannot be adequately understood, or ethically addressed, as a problem of individual parental choice.

### Situating the findings within the literature

The principlist framework (7), while indispensable for mapping the ethical landscape, proves insufficient when applied to the complexities revealed by this study. The principle of respect for autonomy, for instance, presupposes a rational agent with access to adequate information and freedom from coercion. Yet our data show that these conditions are frequently absent: parents receive inadequate information from healthcare providers, encounter systematic misinformation on social media, and, in some families, lack the social power to make independent decisions. When a young mother defers to her mother-in-law’s opposition to vaccination, or when a father’s religious convictions override a mother’s wish to vaccinate, the language of “parental autonomy” obscures more than it reveals.

This finding aligns with the relational autonomy framework developed by Sherwin (29) and Mackenzie and Stoljar (30), which insists that autonomy is not an individual property but a relational capacity, enabled or constrained by social structures, power relations, and access to epistemic resources. Applied to the vaccine refusal context, relational autonomy directs attention away from the question “did this parent choose freely?” and towards the structural conditions that shape and limit choice. In the Turkish context, these conditions include the performance-based payment system that curtails consultation time, the absence of authoritative religious guidance on vaccine permissibility, the gendered division of decision-making authority within the family, and the largely unregulated online information environment.

The convergence between the two participant groups on the question of information carries a specific ethical implication. The system designed to ensure high vaccination coverage may itself undermine the conditions necessary for informed decision-making. When healthcare workers lack the time to counsel hesitant parents, and parents turn to unregulated online communities for information, the resulting refusal cannot straightforwardly be characterized as autonomous in the sense required by bioethical frameworks of informed consent (7). The information asymmetry identified here is therefore not simply a gap in knowledge but a systemic condition with ethical consequences. As Bester (39) has argued, education alone cannot resolve vaccine refusal when the clinical relationship itself is under strain.

The two levels at which participants located their distrust, the institutional and the interpersonal, correspond to a distinction that has since been established empirically. Krastev et al. (40) found institutional distrust, rather than interpersonal distrust, to be the stronger predictor of vaccine hesitancy, and our data suggest that in the Turkish context the performance system operates on both levels simultaneously: it incentivizes healthcare workers to prioritize documentation over dialogue, and it supplies parents with a concrete referent for the suspicion that vaccination serves financial rather than health interests. The trust findings also resonate with Attwell et al. (24) analysis of vaccine-rejecting parents’ engagement with expert systems in Australia. Like their Australian participants, the Turkish parents in our study perceived the pharmaceutical industry as exercising a pernicious influence over healthcare, generating a structural distrust that individual clinicians could not easily overcome. However, our study adds a dimension largely absent from the Australian context: a visible financial mechanism linking the clinician in front of the parent to the coverage targets of the system behind them. This finding has implications for the foundational role of trust in the clinical relationship, as articulated by Pellegrino and Thomasma (41), and supports Goldenberg’s (42) argument that vaccine hesitancy is fundamentally a crisis of public trust rather than a deficit of individual knowledge. The interaction between parents and healthcare professionals is a critical site where trust is built or eroded (25).

It is worth noting that the healthcare workers’ scepticism about reported coverage figures, namely the observation that rates “always appear as one hundred per cent”, has since received administrative confirmation. Until 2024, family physicians in Türkiye could record a missed dose in a way that removed it from the performance metrics used to calculate pay deductions, with the consequence that many refusals never reached the national surveillance database; when documentation was made mandatory in May 2024, recorded refusal rates in one province rose from 3.9 to 21.6 per 1,000, a shift attributed to prior under-ascertainment rather than to a genuine escalation (11). Our participants described in 2017 the mechanism whose magnitude was quantified seven years later.

The religious dimension of vaccine refusal in Türkiye presents ethical challenges that are both locally specific and globally relevant. Our participants’ concerns about vaccine ingredients echo findings from Muslim communities across Southeast Asia, the Middle East, and Europe (43). The ethical challenge posed by religiously motivated refusal is considerable: it requires healthcare professionals to navigate between respect for religious freedom and the imperative to protect children’s health (44). At the time of data collection, no authoritative religious body in Türkiye had issued a formal statement on the permissibility of childhood vaccines, leaving a normative vacuum that was filled by informal religious figures and faith-based online platforms, a pattern consistent with our participants’ accounts of where their information originated.

The contrasting patterns in the fourth theme raise a fundamental question for bioethics: whose autonomy does “parental autonomy” actually represent? In families where the effective decision-maker is a patriarch or a mother-in-law rather than the parent who provides primary care, the language of autonomous choice obscures power relations that may compromise the child’s best interest. As Sherwin (29) and Mackenzie and Stoljar (30) have argued, autonomy is not an individual attribute exercised in isolation but a relational capacity shaped by social structures, interpersonal dynamics, and access to resources. Recent research has similarly demonstrated that vaccine decision-making is deeply gendered, with mothers bearing disproportionate responsibility for what McKenzie et al. (45) term vaccine “decision work.” In the Turkish context, the gendered and intergenerational power structures that govern vaccine decision-making demand a bioethical analysis that moves beyond the individualist model of autonomy towards a relational understanding.

### Differing tractability of the four tensions

The four tensions may not be equally amenable to intervention. Information asymmetry and, to a lesser extent, institutional distrust can be addressed through instruments public health already possesses: longer consultations, clearer communication, and more transparent regulation. Refusal grounded in religious conviction or shaped by family authority appears less tractable, and it is worth considering why. Amin et al. (46) report that vaccine hesitancy is associated less with the moral foundations of harm and fairness, to which public health messaging typically appeals, than with those of purity and liberty. This may help to explain our participants’ accounts, in which the objection concerned the perceived content of vaccines rather than their effectiveness, as well as the observation by healthcare workers that religiously motivated refusal was the most difficult to address. It is also consistent with evidence that corrective information does not reliably increase intention to vaccinate (47).

This does not imply that such concerns cannot be engaged. Reviews of religious teachings on vaccination indicate that questions of this kind, including the transformation of ingredients during manufacture, dilution, and the distinction between medicinal and dietary use, have been considered within several faith traditions (48). Engaging them appropriately, however, calls for resources beyond clinical training, which suggests a role for religious literacy training and for guidance from recognized religious authorities. Family authority structures present a different difficulty, since the person the health system reaches may not be the person who decides. A scoping review of 92 studies across 43 countries identified women’s limited decision-making autonomy as the most frequently reported gender-related barrier to immunization coverage (49), and our participants described comparable situations. Where this is the case, counselling directed only at the parent who attends the clinic may have limited effect, which suggests a need for clinical approaches that take account of how decisions are distributed within the household.

### Pre-COVID-19 baseline and contemporary relevance

These data were collected in 2017, before the COVID-19 pandemic transformed public discourse on vaccination globally. The pandemic amplified many of the dynamics identified in our pre-pandemic data: distrust in healthcare institutions intensified, social media misinformation proliferated at an unprecedented scale, and vaccination became politically polarized in ways that were largely absent from the childhood vaccine debate (26, 27). Recent post-pandemic studies from Türkiye confirm that the themes identified here, institutional distrust, susceptibility to online misinformation, religious and cultural motivations, have persisted and, in some cases, intensified (17, 19). The continuity of these themes across the pandemic divide is itself an important finding: it suggests that the structural and cultural roots of vaccine refusal are deep and enduring rather than merely reactive to specific events or controversies. The subsequent administrative evidence that refusal had been substantially under-recorded within the performance system throughout this period (11) further supports reading the 2017 accounts as documentation of a persistent structural condition rather than of a transient episode.

### Implications for policy and practice

Several implications emerge from these findings. Türkiye, as a signatory to the Council of Europe’s Convention on Human Rights and Biomedicine (1997, ratified 2003), has an international obligation to balance individual rights with the protection of public health. Article 26 of the Convention permits proportionate restrictions on individual rights when necessary for public safety and public health (50). This legal framework underscores the legitimacy of vaccination programs while simultaneously demanding that they respect human dignity and democratic values. First, information provision must move beyond one-directional education towards genuine dialogue. Evidence from behavioral science indicates that motivational interviewing techniques, which emphasize empathic listening and shared decision-making rather than directive persuasion, can improve vaccine coverage relative to conventional didactic approaches (51). Our data suggest that the problem is not merely a deficit of knowledge but a deficit of communicative conditions: healthcare workers need adequate time, training, and institutional support to engage meaningfully with hesitant parents. Reforming the performance-based payment model to incentivize quality of counseling rather than vaccination numbers alone would address a structural contributor to both information asymmetry and trust erosion. Specifically, extending consultation times for vaccine-hesitant families and incorporating measures of counselling quality alongside coverage targets could help realign institutional incentives with patient-centered care.

Second, religious literacy training for healthcare workers could equip clinicians to engage respectfully and knowledgeably with religiously motivated refusal, not to challenge religious beliefs but to identify shared values and resources within religious traditions that support the protection of children’s health (48).

Third, gender-sensitive clinical approaches are needed. In families where patriarchal authority structures marginalize mothers’ voices, healthcare workers may need to create opportunities for private consultation with the primary caregiver, recognizing that the person who presents at the clinic may not be the person who makes the decision (49).

Finally, collaboration with trusted religious and community leaders who support vaccination could help address the normative vacuum identified in this study, providing authoritative guidance that parents currently seek from informal and often unreliable sources. It should be noted here that Türkiye is a modern, constitutionally secular state. Nevertheless, vaccine refusal or hesitancy can still be observed among individuals susceptible to the influence of certain informal religious communities, which hold limited sway within society at large.

### Limitations

Several limitations should be acknowledged. Data were collected in 2017, and the Turkish vaccination landscape has since been reshaped by the COVID-19 pandemic. Notably, during the pandemic period, the Presidency of Religious Affairs in Türkiye issued formal statements supporting childhood vaccination (2020) and endorsing vaccination more broadly as compatible with Islamic practice (2021), partially addressing the normative vacuum identified in this study. Whether these institutional statements have meaningfully reduced religiously motivated vaccine refusal remains an empirical question for future research.

Refusal status among parent participants was established by self-report rather than by verification against immunization records. Some participants had signed a formal refusal form and others had not, and this distinction was not used in analysis; nor was the distinction between partial and complete refusal, which emerged during interviews rather than at recruitment. The findings therefore describe vaccine refusal as a self-identified position and cannot speak to differences between formally documented and undocumented refusers, or between parents who decline all vaccines and those who decline some.

Sampling was criterion-based rather than designed for maximum variation, and no *a priori* quotas were set on education, age, or grounds for refusal. The diversity present in the sample emerged from the referral chains rather than from deliberate selection, and it is possible that snowball recruitment produced clustering around particular social networks or shared rationales for refusal.

Recruitment was concentrated in Istanbul, with three healthcare worker participants from Bursa and no parent participants from outside Istanbul. No comparison between provinces was undertaken, and the sample cannot capture the full diversity of vaccine refusal motivations across Türkiye’s varied regions; healthcare workers’ observations about regional differences, such as those reported in Table 1, are accounts of their experience elsewhere rather than data collected in those settings. At the same time, these features reflect the exploratory, in-depth character of qualitative inquiry rather than flaws of design: the study does not aim at statistical generalization or inter-provincial comparison, and snowball sampling through trusted referral chains was precisely what made a hard-to-reach and often distrustful population accessible.

Finally, the shift from descriptive thematic analysis to normative ethical interpretation was applied retrospectively during the preparation of this article, rather than being built into the original research design; this introduces an interpretive layer that was not anticipated during data collection. While we have argued that the pre-pandemic timing constitutes a distinctive contribution, the findings should be interpreted in their historical context and their contemporary relevance assessed through comparison with post-pandemic research.

## Conclusion

This study has explored the ethical dimensions of childhood vaccine refusal in Türkiye through the perspectives of both vaccine-refusing parents and vaccine-administering healthcare workers, by means of a qualitative study design. The dual-perspective design reveals a central finding: both parents and healthcare workers are caught within structural and relational constraints that compromise the conditions for authentic autonomous decision-making. Parents who refuse vaccination do so within an information environment shaped by inadequate clinical counseling, unregulated online misinformation, informal religious actors who discourage vaccination, and family power structures that may silence the primary caregiver. Healthcare workers who administer vaccines operate within a performance-driven system that prioritizes documentation over dialogue, eroding the very trust upon which vaccination programs depend.

The four themes identified, information asymmetry, the crisis of trust, the conflict between religious and medical authority, and contested family autonomy are not problems that can be resolved through better information provision alone. They demand structural reform, interprofessional collaboration, and an analytical framework that moves beyond individual autonomy towards a relational understanding of how health decisions are made within families, communities, and institutions. The analysis further suggests that these tensions may not be equally tractable: the first two lie largely within the reach of health system reform, whereas the latter two involve normative and social structures that the health system does not control and may need to work with rather than against.

As the post-pandemic world confronts ongoing challenges to vaccine confidence, these pre-COVID-19 findings serve as a reminder that the roots of vaccine refusal are deep, cultural, and ethical, shaped by structures of power, trust, and authority that long predate any single disease outbreak. Effective vaccination programs require not only scientific evidence and logistical efficiency but also genuine engagement with the lived realities of the communities they serve. Public health policies and strategies grounded in the ethical principles of weighing harms and benefits, the best interests of the child, and the prevention of harm to others should attend to the structural determinants of vaccine refusal, including trust in institutions. Last but not least, approaches that consider gender equality, rely on health communication without compulsion, and interact with culturally responsive groups are more likely to achieve sustainable improvements in vaccination coverage than information campaigns alone.

## Data Availability

The datasets presented in this article are not readily available because the qualitative datasets generated and analysed during the current study are not publicly available due to participant confidentiality, as the interview transcripts contain sensitive personal information that could potentially identify participants. Anonymised excerpts are available from the corresponding author on reasonable request. Requests to access the datasets should be directed to Esra Sezer, esra.sezer@acibadem.edu.tr.
